# Modulation of Gene Expression in a Sterile Atopic Dermatitis Model and Inhibition of *Staphylococcus aureus* Adhesion by Fucoidan

**DOI:** 10.3390/dermatopathology8020012

**Published:** 2021-03-25

**Authors:** Ah Young Park, Maureen Bourtembourg, Aline Chrétien, Roland Hubaux, Céline Lancelot, Michel Salmon, J. Helen Fitton

**Affiliations:** 1 Marinova Pty Ltd., 249 Kennedy Drive, Cambridge, TAS 7170, Australia; 2 StratiCELL, Crealys Science Park, Rue Jean Sonet 10, B-5032 Isnes, Belgium; mbourtembourg@straticell.com (M.B.); achretien@straticell.com (A.C.); rhubaux@straticell.com (R.H.); clancelot@straticell.com (C.L.); msalmon@straticell.com (M.S.)

**Keywords:** atopic dermatitis, *Staphylococcus aureus*, fucoidan, periostin, reconstructed human epidermis

## Abstract

Atopic dermatitis is a multifactorial pathology that includes perturbations of gene expression and increased adhesion of *Staphylococcus aureus.* Fucoidans are seaweed-derived sulfated fucose-rich polysaccharides that are known to be anti-inflammatory and may inhibit adhesion of pathogens. Fucoidan was assessed for effects on gene expression of an in vitro 3D model of atopic dermatitis. It was also assessed for inhibitory effects on the adhesion of bacteria onto 3D reconstructed skin. Fucoidan significantly altered gene expression in the atopic dermatitis model, and there was a trend to reduce periostin levels. Fucoidan significantly inhibited the adhesion of *Staphylococcus aureus* and *Cutibacterium acnes* but did not affect the adhesion of *Staphylococcus epidermidis*. Fucoidan may be a useful topical agent to assist in the management of atopic dermatitis.

## 1. Introduction

Atopic dermatitis (AD) is a multifactorial debilitating skin condition that is associated with the bacteria *Staphylococcus aureus,* and alterations in the expression of genes involved in barrier function, itch and inflammation. A combination of environmental exposure, epidermal barrier disruption and immune dysregulation are all part of this condition, which includes severe pruritus and eczematous changes. Bacterial involvement may not be an initiator of the condition, but a sequel to it. Treatment of atopic dermatitis may involve both topical and systemic steroids, methotrexate, biological immunomodulators and antibiotics. The disease burden of AD is considerable, affecting people of all ages and ethnicities [1], and there is an unmet need for effective, non-toxic treatments.

Fucoidans are brown seaweed-derived sulfated fucose-rich polysaccharides that are known to be anti-inflammatory [2,3] and may inhibit the adhesion of pathogens [4,5]. Fucoidan is classically known as a selectin and scavenger receptor blocking agent [6]. By blocking these cellular adhesion molecules, fucoidan can prevent the intrusion of neutrophils into tissue spaces, attenuating inflammatory responses. Pharmaceutical agents that target a specific pro-inflammatory pathway in AD have been greatly explored [7]. Fucoidan has significantly reduced the elevated level of IgE in human peripheral blood mononuclear cells of AD patients in vitro [8] and topical fucoidan has been shown to ameliorate AD in a mouse model [9,10]. Despite these beneficial effects of fucoidan in AD, it is not known how fucoidan affects human epidermal keratinocytes and its gene expression.

Reconstructed human epidermis (RHE) provides a great in vitro research platform, since it mimics layering, differentiation and barrier function of normal human in vivo epidermis [11]. Keratinocytes form the outermost layer of the skin and constitute 90% of epidermal skin cells. When pathogens are introduced onto the surface of skin, keratinocytes begin to produce pro-inflammatory mediators, leading to phenotype features of AD [12]. AD is characterized by an imbalance of the Th1/Th2 and during the acute phase of the disease, Th2-mediated cytokines such as interleukin-4, -13 and-25 are overly expressed [13]. Introduction to these interleukins to RHE induces histological and gene expressions changes typical to the AD patients [14].

In this research, we sought to understand how fucoidan might affect gene expression and periostin production in an in vitro 3D-RHE of keratinocyte model of atopic dermatitis using Th2-mediated cytokines. Periostin is significantly overexpressed in AD patients [15,16,17,18] and in our Th2 cytokine stimulated model [14,19]. We then explored how fucoidan could affect the adhesion of three types of human skin bacteria—the pathogenic *Staphylococcus aureus,* commensal *Staphylococcus epidermidis* and *Cutibacterium acne.*

## 2. Methods and Materials

### 2.1. Materials

Fucoidan extracts from *Fucus versiculosus* (FV) and *Undaria pinnatifida* (UP) were provided by Marinova Pty Ltd. (Cambridge, Australia). The proprietary aqueous extract was designed for topical application and specific properties are described in Table 1. 

### 2.2. Reconstructed Human Epidermis—RHE

The study was carried out on epidermis in vitro, reconstituted with NHEKs keratinocytes isolated from foreskin of 3 Caucasian donors (RHE). The tissues were cultured at the air–liquid interface, during 14 days, in Epilife medium (Fisher Scientific, Merelbeke, Belgium, M-EPI-500-A) containing supplements and antibiotics (Gentamycin, Fisher Scientific, 15710-049). They were maintained in a humid atmosphere at 37 °C with 5% CO_2_. 

### 2.3. Induction of the Th2-Inflamed Model 

Three Th2-related cytokines (IL-4, IL-13 and IL-25) interleukins were applied on the culture medium of RHE at the concentrations of 50 ng/mL for IL-4 and IL-13, and 20 ng/mL for IL-25, during 48 h to induce alterations and gene expression modulations reminiscent to AD and a sensitive skin [14,20,21,22].

### 2.4. Treatment of the Epidermis—Gene Expression

FV and UP were diluted in PBS at 100 μg/mL and applied topically on RHE during the 48 h of stimulation. A mesh has been applied on the *stratum corneum* to allow the even distribution of testing compounds on the tissue surface. For control PBS had been applied topically on Th2-stimulated RHE, with addition of a mesh. 

As a reference treatment GW3965 (10 μM) was applied in the culture medium during the same 48 h in combination with the cytokines. GW3965 is an LXR agonist that modulates the expression of genes involved in lipid homeostasis and inflammation with therapeutic potential in AD [14,23,24]. This condition was compared to the corresponding control condition, treated only with DMSO at 0.05%, given the DMSO was used for the preparation of GW3965 in the culture medium.

### 2.5. Analysis of Tissue Morphology 

The tissue morphology was analyzed through a haematoxylin/eosin (H/E) staining. At the end of 48 h treatment, the tissues were fixed in 4% formaldehyde solution, dehydrated and embedded in paraffin. Sections of 6 μm thick of tissue were generated using Leica microtome RM2245 and laid over microscopic slides before staining with H/E used to visualize general tissue morphology. Slides were mounted with specific medium and examined with a Leica DM2000 photomicroscope coupled to a digital camera (Zeiss DFC420C).

### 2.6. Analysis of Gene Expression Modifications 

#### 2.6.1. Total RNA Extraction and Integrity Analysis

At the end of the treatment, total RNAs were extracted using the Qiagen RNeasy kit (Qiagen; 74106). RHE were rinsed with cold PBS and lysed in the ad hoc buffer from the kit. Extraction was performed according to the Manufacturer’s instructions. The integrity of RNA was analyzed and described in Supplementary information (Figure S1). The collected RNAs were stored at −80 °C. 

#### 2.6.2. cDNA Synthesis 

Reverse transcription was performed with the high capacity RNA-to-cDNA kit (Applied Biosystems, Aalst, Belgium; 438706) from total RNA and the cDNAs were then stored at −20 °C until use in polymerase chain reactions. 

#### 2.6.3. TaqMan Assays 

The microfluidic TaqMan qPCR arrays were designed by StratiCELL and manufactured on demand by Applied Biosystems. Data on 96 genes of interest including 3 internal controls Carbonic anhydrase (CA2), Involucrin (IVL) and Loricirin (LOR), and 1 housekeeping gene for normalization purpose, B2M (β2-microglobuline), were gathered from the RHE samples under different conditions (Figure S2). The TaqMan arrays were processed as described by the manufacturer’s instructions (Micro Fluidic Card Getting Started Guide, Applied Biosystems). 

Briefly, 4 μL (4 ng) of cDNA were mixed with 10 μL of TaqMan Fast Advanced Master Mix (Applied Biosystems), 1 μL of TaqMan Gene Expression Assay and 5 μL of RNAse free water before being injected into the arrays and dispersed into the wells by centrifugation. Arrays were sealed and qPCRs were run using the Quantstudio7 Real-Time PCR System (Applied Biosystems) and its software (QuantStudio real time PCR Software v1.3., Applied Biosystems). The thermal cycles were programmed with one first denaturation step at 95 °C for 20 s. The amplification protocol was followed with 40 cycles (1 s at 95 °C and 20 s at 60 °C). 

All the measurements were performed from triplicates of culture (n = 3) and threshold cycles (C_T_) were obtained for each gene. The relative expression levels were calculated by the comparative C_T_ (ΔΔC_T_) method through a combination of statistical analysis [25,26]. Briefly, C_T_ obtained from treated condition was compared with C_T_ obtained the reference condition. This average C_T_ has been normalized to C_T_ of B2M, the reference gene. The relative quantification (RQ) is obtained using the formula below:

RQ = 2^−(ΔC^_T_^treated condition − ΔC^_T_^reference condition)^

where ΔC_T_ = C_T_[target gene] − C_T_[reference gene] in cDNA sample.

The genes for which the C_T_ values were >36 have been considered as non-expressed and omitted of the analysis. These include CNR1, IL6, LCE3B and SEMA3A. 

In order to normalize the results, B2M was amplified from the same cDNA samples. A control without cDNA was performed in parallel as negative control of amplification to verify the absence of contaminants. 

The data obtained for the condition treated with FV and UP were compared to the untreated condition stimulated by Th-2 cytokines. The maximum C_T_ cut-off value was fixed at 36 cycles. 

### 2.7. Analysis of Extracellular Periostin Abundance

The quantifications of the extracellular releases of Periostin (Thermo Scientific, EHPOSTN) were performed on the supernatants of all conditions in a specific Elisa assay. 

### 2.8. Bacterial Cultures, Adhesion of Bacteria, and CFU Counting 

#### 2.8.1. Bacterial Cultures of *Staphylococcus epidermis* and *Staphylococcus aureus*

*Staphylococcus aureus* (ATCC 6538) and *Staphylococcus epidermidis* (ATCC 12228) were obtained from the American Type Culture Collection. The strains were grown in TSB (Tryptic Soy Broth) at 37 °C under 150 rpm and on TSA (Tryptic Soy Agar) plate. 

The overnight (O/N) cultures of *S. epidermidis* and *S. aureus* were prepared in 10 mL of TSB medium from a fresh colony obtained by culturing *S. epidermidis* and *S. aureus* on TSA agar. The resulting cultures were then centrifuged for 10 min at 7000× *g* to harvest the cells and the pellets were re-suspended in 10 mL of PBS. The suspensions were then diluted 1000 fold in order to obtain an inoculum of approximately 10^5^ CFU/mL.

#### 2.8.2. Adhesion of Bacteria and CFU Counting

Adhesion of bacteria has been assessed by quantification of colonies on microbiological plates (Colony Forming Unit counting). After 14 days of culture at the air-liquid interface, RHE surfaces were put into contact for 30 min with 100 μL of FV and UP at 100 μg/mL or 100 μL of PBS for control samples. After 30 min, 100 μL of the bacterial suspensions were added on the RHE surface at a concentration of 10^5^ CFU/mL for 1 h. Control samples were treated with 100 μL PBS. After the contact period, non-adherent cells were eliminated by six consecutive rinses with 300 μL of sterile PBS. Finally, the adherent bacteria were collected in PBS with 0.1% Tween80 with a swab. Both adherent and non-adherent cells were enumerated by seeding on TS agar (according to the decimal dilution method).

## 3. Results

### 3.1. Effects of the Test Compounds on the Morphology of RHE When Cultured under Conditions Mimicking Atopic Dermatitis 

**Figure 1:**
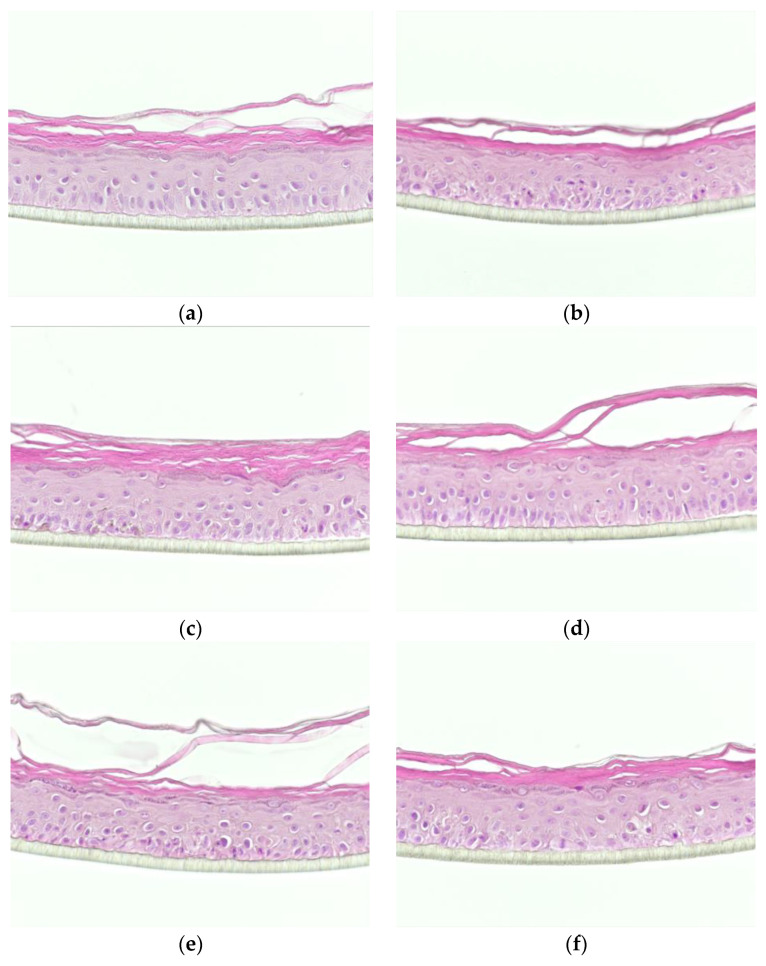
Histological sections of Th2-stimulated reconstructed human epidermis (RHE) after 48 h or topical and systemic treatments with the test compounds and reference of its solvent, respectively (H/E staining; n = 1). (**a**) control; (**b**) Th2 (+PBS topic); (**c**) Th2 + DMSO 0.05% (**d**) Th2 + 10 µM GW3965 in 0.05% DMSO; (**e**) Th2 + 100 µ/mL FV; (**f**) Th2 + 100 µ/mL UP.

Morphology of epidermis was examined after 48 h of topical treatment of FV and UP by H/E staining. This was compared to RHE in the presence and absence of the GW3965 for 48 h (Figure 1). RHE controls (Figure 1a) presented a full differentiation of keratinocytes from basal to cornified layers resulting in the formation of the four typical layers of the epidermis, namely, basal, spinous, granular and cornified layers.

The epidermal incubation with the mix of IL-4, IL-13 and IL-25 influenced the tissue organization, indicating the tissue response to the inflamed environment (Figure 1b,c). This was especially observed in the deepest layers of the RHE and more so, the basal layer, with a loss of polarized orientation of the apical–basal axis and reduced cohesion. As expected, compared to PBS (Figure 1c) DMSO (Figure 1b) did not influence any structural changes to Th2 stimulated tissue organization.

When the reference compound GW3965 was applied to the RHE, the morphological changes induced by the Th2 stimulation were considerably reduced, prohibiting the alteration of the polarized orientation of the basal layer (Figure 1d). As expected, DMSO alone did not change the morphology of the Th2–induced RHE, indicating that the GW3965 compound itself is responsible for reducing morphology change brought by cytokines (Figure 1c). The topical treatment with 100 μg/mL of FV and UP on Th2-induced RHE did not lead to additional morphological changes however no cytotoxicity was observed (Figure 1e,f). This supports the non-noxious impact of FV and UP on reconstructed epidermis.

### 3.2. Gene Expression Analysis by RT-qPCR Using TaqMan Array

To better understand whether topical treatment of FV and UP alters any AD related gene expressions, we analyzed 93 target genes that are specifically designed to assimilate in vitro AD environment. 

**Figure 2:**
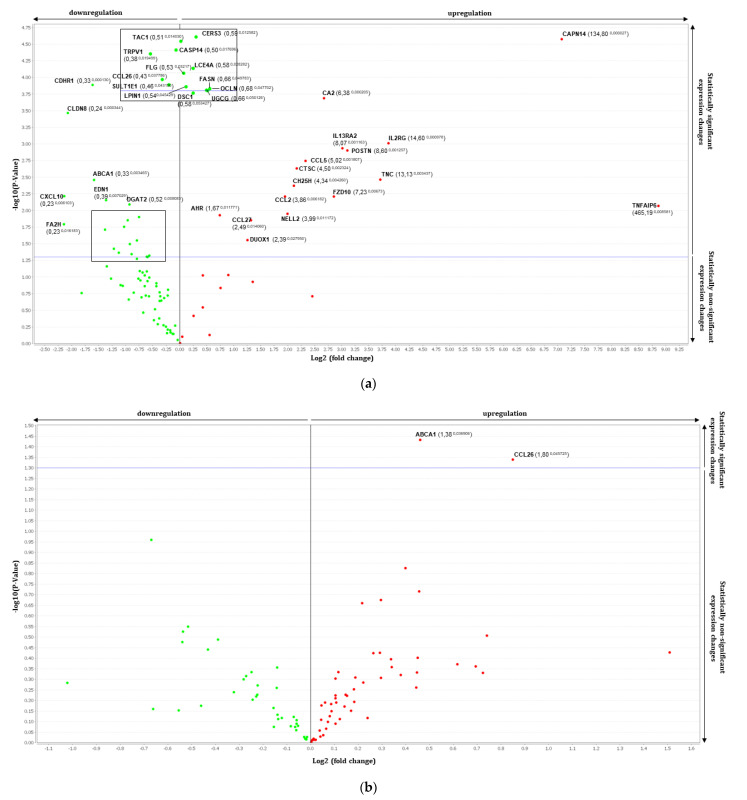

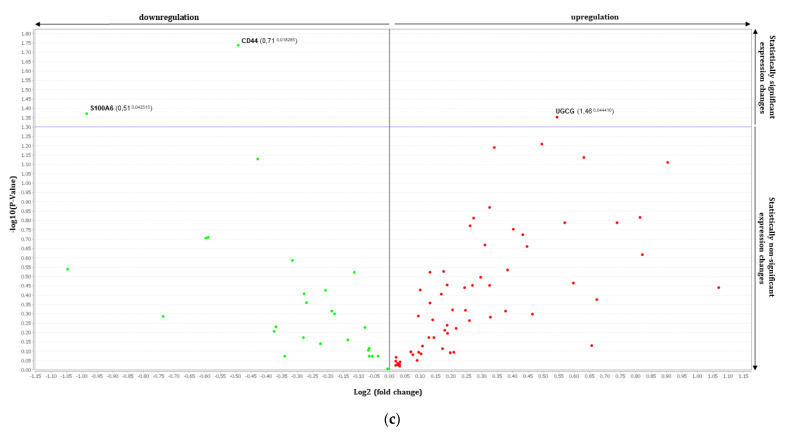
Volcano plots in analyzing differential gene expressions comparing between (**a**) control RHE and Th2 induced-RHE, (**b**) Th2 induced-RHE and FV treated Th2 induced-RHE and (**c**) Th2 induced-RHE and UP treated Th2 induced-RHE. Fold change boundary: 1 and *p*-value boundary: 0.05.

First, to validate that introduction of Th2 related cytokines (IL-4, IL-13, and IL-25) can stimulate atopic dermatitis environment, we looked at differentially expressed genes with *p*-value < 0.005. A large number of differentially expressed genes in Th2-induced RHE were observed compared to the normal RHE environment as expected (Table 2). A global analysis of the differentially regulated genes by Th2 stimulation was carried out and volcano plot was used to highlight significant variations of gene expression (Figure 2a). 

Four genes that prompted most fold changes were TNFAIP6 (tumor necrosis factor-inducible gene 6 protein), CAPN14 (Calpain-14), IL2RG (IL-2 receptor subunit gamma) and TNC (Tenascin-C). These genes are all up regulated in Th2-induced RHE and are inflammatory mediators. Moreover, NELL2 (neural epidermal growth factor-like 2) and CA2 (carbonic anhydrase 2) that are representative of atopic dermatitis and differentiates from psoriasis were also upregulated. 

Down-regulated genes are OCLN, FASN, UGCG and CES3 mainly involved in barrier recovery and lipid homeostasis. 

Both topical treatments of FV and UP to Th2 induced-RHE induced significant changes in the expression of a few genes compared to the stimulation alone (Table 2 and Figure 2b,c). In the presence of FV, CD44 (CD44 antigen, hyaluronate receptor) and S100A6 (S100 calcium binding protein A6) were down regulated, whereas UGCG (Ceramide glucosyltransferase) was upregulated. There were many non-significant modifications and this concerns especially the regulation of genes involved in lipid homeostasis and barrier recovery (Table 3). The majority of them appear to be regulated in an opposite direction than what observed in tissue cultured in presence of Th2 cytokines alone. 

The treatment of Th2 stimulated epidermis with UP tended to reverse a greater number of Th2-related effects on gene expression than found in response to treatment with FV. Indeed, a broader panel of Th2 gene targets were inversely regulated after UP application compared to FV application (Table 3). 

### 3.3. Effects of the Test Compounds on Periostin Release by RHE When Cultured under Conditions Mimicking Atopic Dermatitis 

**Figure 3:**
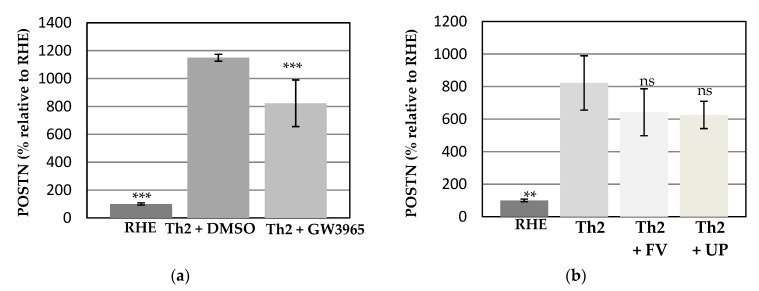
Changes in the periostin (POSTN) abundance in (**a**) Th2-induced RHE in 0.05% DMSO and in the presence of GW3965 and (**b**) in the presence of FV and UP. Data are given in percentage relative to the untreated condition. (*** *p* < 0.001, ** 0.001 < *p* < 0.01 and ns *p* > 0.05).

To investigate whether the FV and UP treatment could modulate the protein expression of periostin, a well-known maker of AD [15,16,17,18], the analysis was performed by a specific immunoassay (ELISA) targeting periostin. The changes of periostin production are shown in Figure 3a,b. 

The Th2 stimulation (+DMSO) induced a strong and significant production of periostin as compared to unstimulated condition (Figure 3a). As expected, the presence of GW3965 allowed to significantly decrease this induction, favoring a return towards a basal level of protein expression [14]. 

The Th2 challenge combined with topical application of PBS triggered an important and significant release of periostin. It is interesting to note that the induction level of periostin protein expression was found similar to those of its gene expression, supporting the relevance to study this marker in the present model. In a similar way, the addition of FV or UP stimulated a reduced production of periostin shown in Figure 3b. Although not significant, this downward tendency supports a protective effect of FV and UP treatments on the regulation of periostin. 

### 3.4. Adhesion of Bacteria

*Staphylococcus aureus* colonizes the skin of majority of atopic dermatitis patients and it could lead to increase disease severity. To test whether fucoidan could prevent or minimize bacteria adhesion, we evaluated the impact of FV and UP at 100 μg/mL on the adhesion of *Staphylococcus epidermidis*, *Staphylococcus aureus* and *Cutibacterium acnes* on RHE. Adhesion of bacteria has been assessed by quantification of colonies on microbiological plates after collection of adherent and non-adherent cells from the RHE tissues, as explained in the methods.

**Figure 4:**
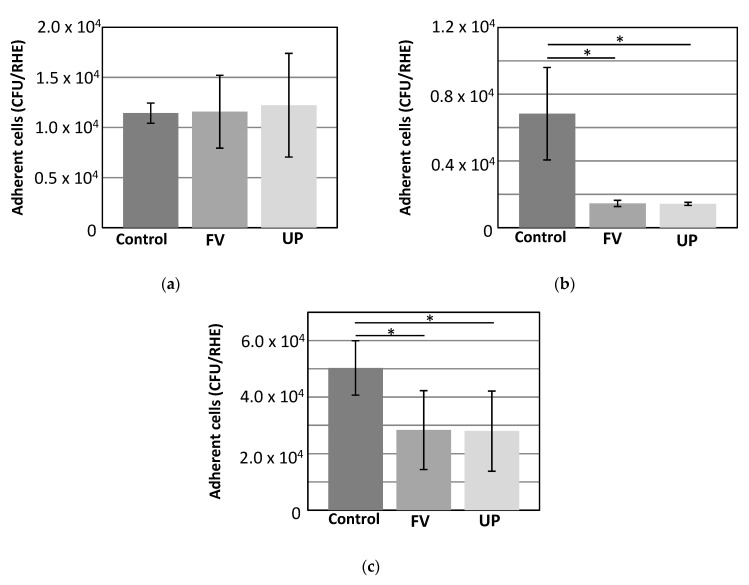
Concentrations of adherent (**a**) *S. epidermidis*, (**b**) *S. aureus* and (**c**) *C. acnes* after topical treatment with FV and UP. The concentrations are expressed in CFU/RHE. The graph represents the means obtained on three independent cultures with three counts for each of them, as well as the standard deviation. The values of 0.001 < *p* < 0.05 are considered as significant (*). Student’s test compared to control.

There was no significant effect neither with FV or UP on the adhesion of the commensal *S. epidermidis* (Figure 4a). However, a significant reduction of *S. aureus* adhesion was observed upon prior topical application of FV and UP on the *stratum corneum* of RHE (Figure 4b). Furthermore, topical application of FV and UP reduces the adhesion of the *C. acnes* (Figure 4c). 

## 4. Discussion

Sensitive skin with atopic tendency and atopic eczema are common skin problems/diseases associated with altered epidermal barrier and, for the most severe cases, chronic inflammation. Atopic dermatitis has a strong Th2 component associated with IL-4 and IL-13 over-production by Th2-polarized lymphocytes in the acute phase of the disease. 

The RHE model mimicking an inflammatory context through Th2 cytokines stimulation and used in this study was fully characterized by the demonstration of the dysregulation of many genes involved in skin barrier, lipid metabolism and transport, axon guidance and inflammation. These data are in correlation with other published studies on closely related in vitro models [14,22,27,28].

Both treatments of Th2-induced RHE with FV and UP stimulated significant changes in expression of a few genes compared to the Th2 induction alone. Insoluble lipids require specific transport mechanisms or carriers such as ABCA1 to move them through the cytoplasm [29]. Keratinocytes require abundant cholesterol for cutaneous permeability barrier function, hence the regulation of cholesterol homeostasis is of great importance. ABCA1 is a ubiquitous membrane transporter responsible for cholesterol efflux and plays pivotal role in regulating cellular cholesterol levels. A significant decrease of the ABCA1 expression following Th2 stimulation was restored and increased its expression by 1.38 fold by FV. Therefore, FV considerably improve or stabilize the cholesterol homeostasis, through the up-regulation of ABCA1, in tissues whose barrier function is impaired in context of Th2 inflammation. 

Interestingly, FV also induced the significant gene regulations of CCL26, a chemokine involved in the recruitment of inflammatory cells. The increased expression of the Th2-down-regulated chemokine may support the ability of FV to reverse the Th2 effect, but also promote the recruitment of cells involved in wound healing process [30,31]. 

The differential expression of CD44, the receptor for hyaluronic acid, was also reversed in the presence of UP. Increased expression of the hyaluronic synthase 3 (HAS3) by Th2 stimulation was also decreased in response to FV application. These co-regulated genes may indicate the ability of fucoidan to act on the metabolism of hyaluronic acid and related pathways, and more so, to counteract spongiosis, a hallmark of eczema [32]. In spongiosis, keratinocytes lose cohesiveness with a decreased expression of cadherins, as a water influx into the epidermal intercellular spaces occurs together with an accumulation of hyaluronate (HA). This phenomenon is associated with an increased expression of CD44, the major receptor of HA and HAS3. All these components are known to be up-regulated in lesional AD skin as a consequence to Th2 inflammation [32,33,34,35]. 

S100A6 encodes for a calcium binding protein of the S100 family and is implied in the regulations of the differentiation process of keratinocyte [36] and was down-regulated by UP. The overexpression of this gene of the S100 family in keratinocyte is associated with an undifferentiated status of the cell. Therefore, the great down-regulation of S100A6 by the test compound could demonstrate a promoted differentiation of keratinocyte. 

UGCG participates in the glycosylation of ceramides. One of remarkable and epidermis-specific ceramide species is acylceramides and they are essential for skin barrier formation. Decreases in acylceramide levels as well as alteration in ceramide composition and chain-length are associated with cutaneous disorders such as ichthyosis and psoriasis, and represented a hallmark of AD [37,38]. Upregulation of UGCG by UP could, therefore, have a positive effect on ceramides metabolism in the skin, by a return to normal level and on the restoration of the resulting barrier function in the event of an inflammatory challenge. 

A large number of subtle modifications were also observed (Table 3). The majority of affected genes are involved in lipid homeostasis and barrier recovery and are regulated in an opposite way compared to Th2-induced RHE. Although not significant, all these subtle but concomitant regulations might demonstrate a bundle of evidence which acts in complementarity to generate epidermal benefits against AD-related detrimental effects. Therefore, fucoidan could act in favor of a reinforced and more resistant epidermal barrier, alleviating the damages associated with sensitive skin and AD.

Most importantly fucoidan significantly inhibited adhesion of *S. aureus* which is typically abundant in skins of AD patients and aggravate the condition [39]. However, interestingly fucoidan did not affect the adhesion of *S. epidermidis,* usually innocuous commensal skin bacteria. It is unclear how fucoidan differentiates between *S. aureus* and *S. epidermidis*. As a relatively hydrophobic bacteria, *S. epidermis* was found to adhere to stainless steel more effectively than to reconstituted skin. However, the opposite was true for *S. aureus* which is known to have a basic and hydrophilic surface [40]. We speculate that negatively charged fucoidan binds to *S. aureus* thereby preventing it to adhere to the RHE in this study. 

Interestingly, antimicrobial activity against *E. coli* and *S. aureus* of fucoidan has been observed before [41] and showed a great potential as an antiadhesive biomaterial coating agent that stops infection of *S. aureus* for urinary catheter applications [42].

Acne is a common skin disease which affects 9.4% of the global population [43] and over-colonization of *Cutibacterium acnes* is one of the main triggers for acne. Interestingly, recent studies indicated that the balance of *C. acnes* and *S. epidermidis* population on skin is very important as *S. epidermidis* can limit *C. acnes* over-colonization [44]. Considerable inhibition of adhesion of *C. acnes* and maintenance of *S. epidermidis* population may potentiate fucoidan as a beneficial therapeutic agent in acne treatment.

## 5. Conclusions

Topical application of fucoidan from *Fucus versiculosus* and *Undaria pinnatifida* leads to non-cytotoxic impact on the 3D reconstructed human epidermis. Together with beneficial gene regulation, subtle inhibition of periostin release and remarkable inhibition of *S. aureus* adhesion, fucoidan offers a promising therapeutic potential against skin inflammatory disease such as atopic dermatitis and related damages.

## Figures and Tables

**Table 1:** Description of *Fucus versiculosus* (FV) and *Undaria pinnatifida* (UP).

Fucoidan Extract	Neutral Carbohydrates	Sulfate	Cations (approx.)	Fucoidan	Polyphenol
FV	43.7%	10.1%	3%	58.6%	33.7%
UP	48.8%	27.4%	9%	89.6%	<2%

**Table 2:** Differentially expressed genes that are shown significant changes. The increases are in green and decreases in red.

**Th2-Induced RHE vs. RHE**					
**Genes**	**Fold Change**	***p* Value**	**Genes**	**Fold Change**	***p* Value**
ABCA1	0.33	0.0035	AHR	1.67	0.0118
CASP14	0.49	0.0177	CA2	6.38	0.0002
CCL26	0.43	0.0378	CAPN14	134.80	0.0000
CDHR1	0.33	0.0001	CCL2/MCP1	3.86	0.0062
CERS3	0.59	0.0126	CCL27	2.49	0.0141
CLDN8	0.24	0.0003	CCL5/RANTES	5.02	0.0018
CNR1	0.51	0.0346	CH25H	4.34	0.0043
CXCL10	0.23	0.0061	CTSC	4.50	0.0023
DGAT2	0.52	0.0081	DUOX1	2.39	0.0279
EDN1	0.39	0.007	FZD10	7.23	0.0062
FA2H	0.23	0.0162	IL13RA2	8.07	0.0012
FASN	0.66	0.0498	IL2R	14.60	0.001
FLG	0.53	0.0321	NELL2	3.99	0.0112
LCE4A	0.58	0.0283	POSTN	8.60	0.0013
LPIN1	0.54	0.0454	TNC	13.13	0.0034
OCLN	0.68	0.0477	TNFAIP6	465.19	0.0086
SEMA3A	0.51	0.0336			
SULT1E1	0.46	0.0431			
TAC1	0.51	0.014			
TRPV1	0.38	0.0195			
**Th2 Induced RHE vs. FV Treated**			**Th2 Induced RHE vs. UP Treated**		
**Genes**	**Fold Change**	***p* Value**	**Genes**	**Fold Change**	***p* Value**
CD44	0.71	0.0183	ABCA1	1.38	0.0369
S100A6	0.51	0.0425	CCL26	1.80	0.0457
UGCG	1.46	0.0444			

**Table 3:** The list for genes involved in complementary regulations about lipid homeostasis and potential regulations on barrier recovery. The increases and decreases are in green and red, respectively, and the significant *p*-values in bold.

Functions	Genes	Th2 vs NT		Th2 vs Th2 + FV		Th2 vs Th2 + UP	
		Fold Change	*p* Value	Fold Change	*p* Value	Fold Change	*p* Value
Triglyceride synthesis	GPAT3	0.6214	0.0861	1.3189	0.1494	1.3213	0.1769
Fatty acid synthesis	ACACA	0.6756	0.1961	1.2663	0.4387	1.6682	0.1628
	FA2H	0.2267	**0.0162**	1.2280	0.4934	2.0985	0.3626
Cholesterol biosynthesis and transport	ABCA1	0.3335	**0.0035**	1.3755	**0.0369**	1.2663	0.0646
	HMGCS1	0.6389	0.1370	0.8291	0.4831	1.8712	0.0777
	NR1H3	0.7706	0.4183	1.3721	0.1926	1.1837	0.3636
	SULT1E1	0.4577	**0.0431**	0.9815	0.9404	1.7573	0.1530
Ceramide synthesis	CERS3	0.5949	**0.0126**	1.0840	0.4628	1.5486	0.0728
	GBA	0.7193	0.4457	1.3629	0.4651	1.7666	0.2421
	SMPD1	0.7417	0.1241	2.8455	0.3736	1.3497	0.1891
	UGCG	0.6619	0.0501	0.9075	0.4411	1.4580	**0.0444**
AM defense	DEFB4A	0.3933	0.0691	1.3601	0.5485	1.2980	0.4851
Epidermal biology	HAS3	5.4933	0.1958	0.4924	0.5206	0.7897	0.8425
Epidermal junction	CLDN25	1.4695	0.7438	0.6323	0.6919	1.5768	0.7407
	OCLN	0.6770	**0.0477**	1.0514	0.7956	1.2521	0.1352
	TJP1	0.6468	0.1899	1.6713	0.3112	1.3790	0.5035
Cornified envelope precursors	CASP14	0.4896	**0.0177**	0.9058	0.5509	1.3626	0.2183
	LCE2A	0.5544	0.1728	1.6514	0.4677	1.5951	0.4212
	SPRR2A	0.5200	0.2194	1.6174	0.4346	0.8558	0.7255
	ZNF750	0.8382	0.5563	0.9098	0.7737	1.5126	0.3437

## Supplementary Materials

The following are available online at https://www.mdpi.com/2296-3529/8/2/12/s1, Figure S1: Analysis of the integrity of extracted RNA through their electrophoresis profiles, Figure S2: Modifications of the gene expression of 3 targets from reconstructed epidermis after 48 h of treatment with Th2-like cytokines in presence or absence of GW3965.
