# Supplementary material for: Modulation of Gene Expression in a Sterile Atopic Dermatitis Model and Inhibition of Staphylococcus aureus Adhesion by Fucoidan
Source: Dermatopathology (Basel). 2021 Mar 25;8(2):69–83. doi: 10.3390/dermatopathology8020012 (PMC8103255; doi:10.3390/dermatopathology8020012)
Supplement: Supplementary file 1 [file dermatopathology-08-00012-s001.pdf]

Supplementary Material

Modulation of Gene Expression in a Sterile Atopic Dermatitis Model and Inhibition of *Staphylococcus aureus* Adhesion by Fucoidan

Ah Young Park, Maureen Bourtembourg, Aline Chrétien, Roland Hubaux, Céline Lancelot, Michel Salmon and J. Helen Fitton

1. RNA Integrity Analysis

The RNA concentration was determined by spectrophotometric measurement (*QI-Axpert-Qiagen*) and the RNA quality was analysed by capillarity electrophoresis (*Agilent Bioanalyzer 2100–Agilent RNA 6000 Nano Kit, 5067-1511*), by visualization of intact ribosomal RNA bands. For the total RNA from higher eukaryotes, the size of the ribosomal bands should be 1.9 kb for the 18S-RNA and 4.7 kb for the 28S-RNA. The intensity of the band corresponding to the 28S RNA must be greater than the intensity of the band corresponding to the 18S-RNA (left profile, in Figure 2). Small and diffuse bands representing low molecular weight RNAs (tRNA and 5S ribosomal RNA) may be present. Degradation of the RNA will be apparent as a smearing of ribosomal RNA bands and high molecular weight background as shown below (right profile, in Figure S1).

In order to validate the test system, amplification of selected targets (CA2, IVL and LOR) after Th2 stimulation was performed and compared to unstimulated control. Moreover, the effect of the GW3965 was also analyzed to reverse the effect of the Th2 stimulation and compared to its DMSO solvent.

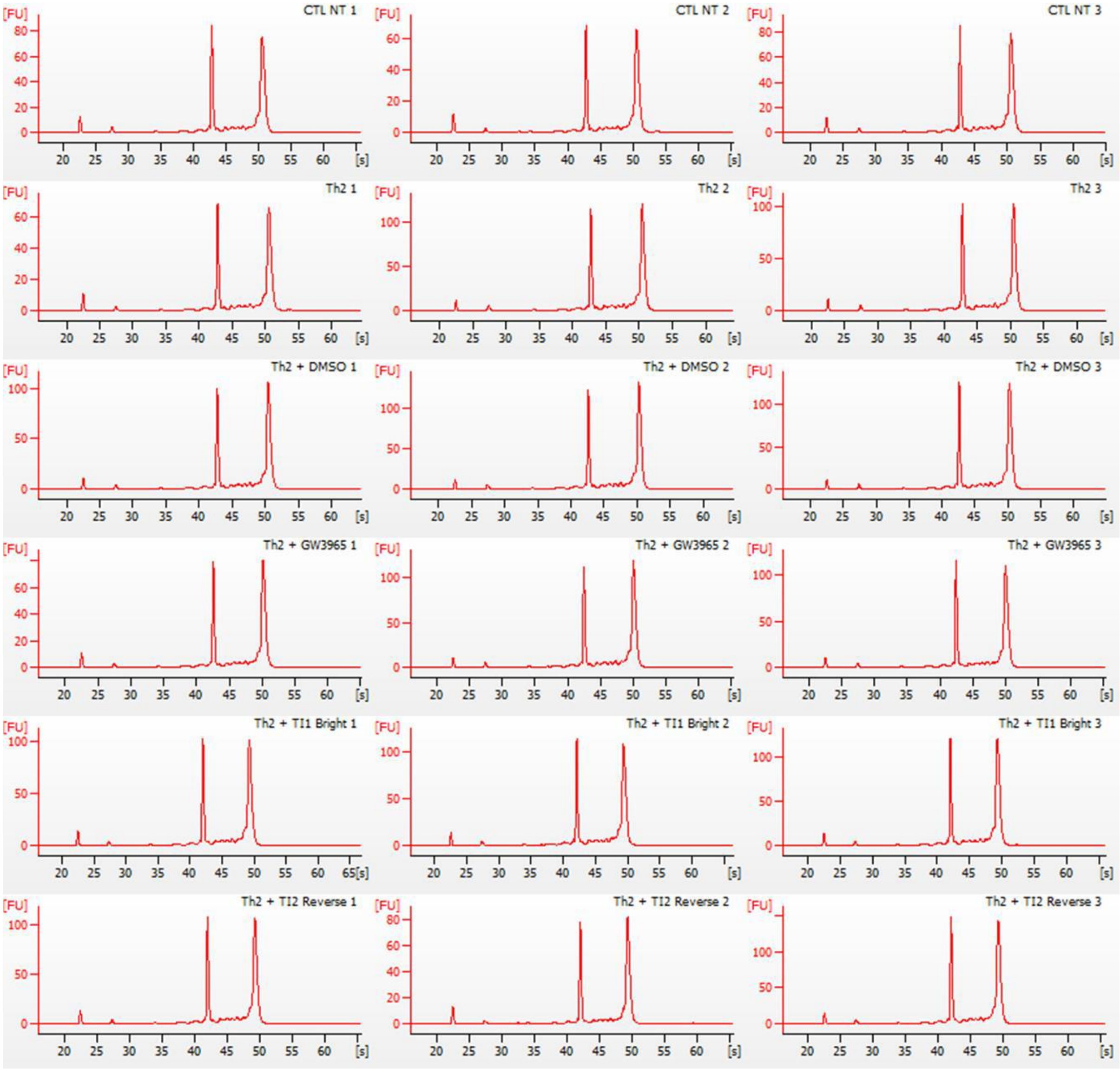

Figure S1. Analysis of the integrity of extracted RNA through their electrophoresis profiles.

2. Test System Validation

The expression profile of three specific targets, Carbonic anhydrase (CA2), Involucrin (IVL) and Loricirin (LOR) was obtained to compare with unstimulated control (Figure S2). The effect of the benchmark molecule (GW3965) was analyzed to reverse the effect of the Th2 stimulation and compared to its DMSO solvent.

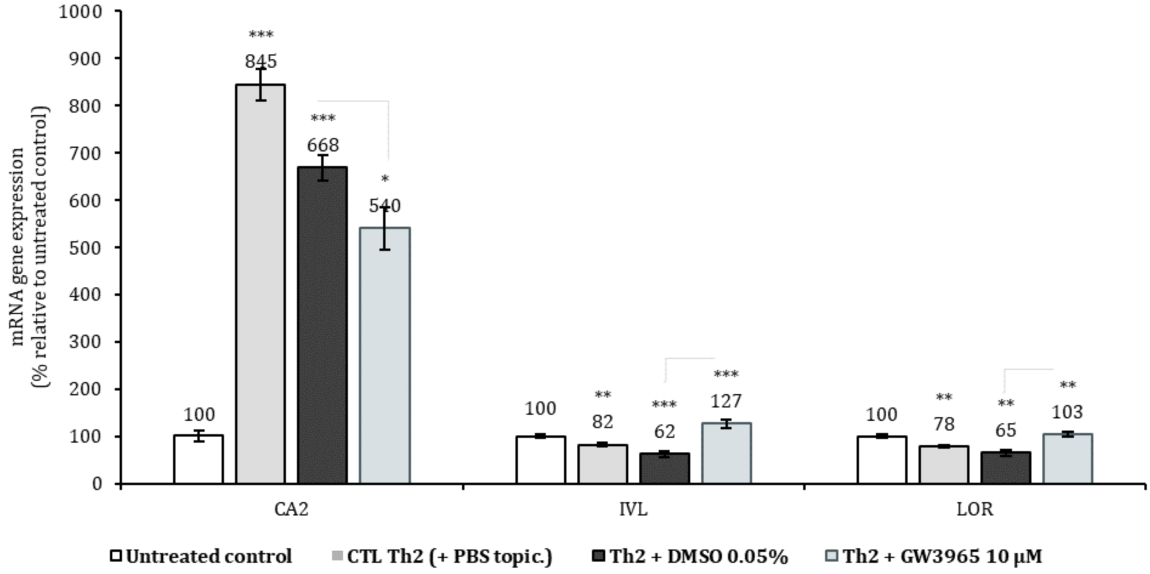

Figure S2. Modifications of the gene expression of 3 targets from reconstructed epidermis after 48h of treatment with TH2-like cytokines in presence or absence of GW3965. Expression data are given in percentage relative to the untreated condition. Mean of data obtained from 3 epidermis per condition ± standard deviations (SD) are shown. Statistical analysis was performed using bilateral Student's *t*-test to compare each Th2-stimulated condition to unstimulated control and the GW3965-treated condition to its specific Th2/solvent control (*** $p < 0.001$; ** $0.001 < p < 0.01$ and * $0.01 < p < 0.05$).

As expected, the Th2 challenge induced a strong and significant up-regulation of CA2, a gene known to be overexpressed in patients with atopic dermatitis, and the significant down-regulations of two genes encoding for key actors of the epidermal differentiation process and barrier formation, involucrin (IVL) and loricrin (LOR). The gene expression profiles were reminiscent to those described in lesional skin of patients with atopic dermatitis, indicating the effective Th2 stimulation to mimic sensitive skin features such as found *in vivo*.

The addition of GW3965 allowed to reverse significantly the Th2-related effects on the expression of the differentiation markers (IVL and LOR) and reduced significantly the Th2-induced up-regulation of CA2, promoting a return to a basal level of expression.

The transcriptional profiles of the three genes confirmed the responsiveness of the responsiveness of the epidermis to Th2 stimulation and the benchmark. These results validate the test system.
